# Pathogen exposure reduces sexual dimorphism in a host’s upper thermal limits

**DOI:** 10.1002/ece3.6828

**Published:** 2020-11-06

**Authors:** Tess Laidlaw, Tobias E. Hector, Carla M. Sgrò, Matthew D. Hall

**Affiliations:** ^1^ School of Biological Sciences and Centre for Geometric Biology Monash University Melbourne Vic. Australia

**Keywords:** aquatic ectotherm, *Daphnia magna*, host–parasite interactions, *Pasteuria ramosa*, population persistence, sex differences, static heat stress, thermal tolerance

## Abstract

The climate is warming at an unprecedented rate, pushing many species toward and beyond the upper temperatures at which they can survive. Global change is also leading to dramatic shifts in the distribution of pathogens. As a result, upper thermal limits and susceptibility to infection should be key determinants of whether populations continue to persist, or instead go extinct. Within a population, however, individuals vary in both their resistance to both heat stress and infection, and their contributions to vital growth rates. No more so is this true than for males and females. Each sex often varies in their response to pathogen exposure, thermal tolerances, and particularly their influence on population growth, owing to the higher parental investment that females typically make in their offspring. To date, the interplay between host sex, infection, and upper thermal limits has been neglected. Here, we explore the response of male and female *Daphnia* to bacterial infection and static heat stress. We find that female *Daphnia*, when uninfected, are much more resistant to static heat stress than males, but that infection negates any advantage that females are afforded. We discuss how the capacity of a population to cope with multiple stressors may be underestimated unless both sexes are considered simultaneously.

## Graphical abstract

Within a population, individuals vary in both their resistance to both heat stress and infection, and their contributions to vital growth rates. No more so is this true than for males and females. Each sex often varies in their response to pathogen exposure, upper thermal limits, and particularly their influence on population growth. We find that females, when uninfected, are much more resistant to static heat stress than males, but that infection negates any advantage that females are afforded.

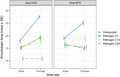

## 1 INTRODUCTION

Global change is resulting in dramatic shifts in both the thermal environment of individual populations and the types of pathogens that might be maintained or arise under these new conditions (Altizer et al., 2013; Lafferty, 2009; Meehl & Tebaldi, 2004). For a population to persist under these pronounced changes, critical thermal limits and susceptibility to infection are, individually, likely determinants of whether the population in its current location can continue to reproduce and thrive, or instead go extinct (Bush et al., 2016; De Castro & Bolker, 2004; Kingsolver et al., 2013; McCallum & Dobson, 1995). Yet, the response of a potential host to thermal stress and infection does not operate in isolation. Exposure to a pathogen has increasingly been shown to influence the thermal tolerance of an organism, usually resulting in a lowering of upper thermal limits (Greenspan et al., 2017; Hector et al., 2019), but within a population individuals will likely differ in their responses, owing to variability in both their susceptibility to infection (Hall et al., 2019; Sheldon & Verhulst, 1996) and tolerance to thermal change (Calosi et al., 2010; Janion‐Scheepers et al., 2018; Sgrò et al., 2010). Understanding population persistence when faced with both global change and infectious disease, therefore, requires linking sources of individual variation to the interplay between infection and thermal tolerances, particularly as not all individuals will contribute equally to the growth of a population (Crowley, 2000; Harts et al., 2014).

One of the greatest sources of variation within a species is the differences that arise between males and females. Each sex is often strikingly different in many aspects of their morphology, reproduction, resistance to infection, and even thermal tolerances (Andersson, 1994; Gipson & Hall, 2016; Lasne et al., 2018; Zuk, 2009). In birds and mammals, for example, males are often more susceptible to infection than females, although the identity of the “sicker sex” depends on the species, particularly for invertebrates (Cousineau & Alizon, 2014; McCurdy et al., 1998; Poulin, 1996; Schalk & Forbes, 1997; Sheridan et al., 2000; Zuk, 2009). Sexual dimorphism is also prevalent in the upper and lower thermal limits of species, with the more tolerant sex again likely to vary across species and broader taxonomic groups (Lasne et al., 2018; Lyons et al., 2012; Mitchell & Hoffmann, 2010). For any species with separate sexes, therefore, males and females are highly likely to vary in both their thermal tolerances and their response to infection, but rarely has the interplay between sex and pathogen exposure been considered in terms of the thermal performance of a population or species.

Sex difference in thermal limits both before and after exposure to a pathogen becomes particularly important when considering population persistence under the dual threat of global change and infectious disease. Population growth is often thought to be closely tied to female fitness alone, owing to both the higher parental investment that females typically make in their offspring and the assumption that female fecundity is independent of male abundance ("female demographic dominance,” Crowley, 2000; Harts et al., 2014). Persistence in the face of global change is thus likely to be a function of female thermal limits alone, at least for species without substantial male parental investment (for a variety of scenarios where males matter see Rankin & Kokko, 2007). But what happens if infection by a pathogen reduces an individual's tolerance to heat stress? If the sex which is most closely tied to population growth (typically females) is more resistant to infection, then a population might be buffered against any reduction in thermal limits caused by infection. Conversely, if population persistence depends on the fitness of the more susceptible sex, then a species will be more vulnerable to thermal stress than previously thought.

In this study, we explore how host sex and exposure to a bacterial pathogen, *Pasteuria ramosa*, interact to influence the upper thermal limits of the water flea, *Daphnia magna*. Previously, using only female hosts, infection by *P. ramosa* has been shown to result in a substantial reduction in the thermal limits of its host under both static and ramped forms of heat stress (Hector et al., 2019). This reduction in both critical thermal maxima (CT max) and time to immobilization (i.e., knockdown times) was on par with the changes in thermal limits commonly observed across large geographical ranges for *Daphnia* (Yampolsky et al., 2014) and other species (Calosi et al., 2010; Hector et al., 2020; Janion‐Scheepers et al., 2018; Sgrò et al., 2010). When considered in light of recent studies showing evidence of local adaptation across *Daphnia* host genotypes (Seefeldt & Ebert, 2019; Yampolsky et al., 2014), and evidence that temperature influences many aspects of pathogen fitness and disease dynamics (Auld & Brand, 2017; Shocket et al., 2018; Vale & Little, 2009), the results of Hector et al. (2019) add to the growing recognition that exposure to thermal stress may influence the ecology and evolution of both host and pathogen species. In all cases, however, only females have been the direct focus of study, as with many other studies of thermal performance (Calosi et al., 2010; Hector et al., 2020; Janion‐Scheepers et al., 2018; Sgrò et al., 2010), and the impact of both thermal stress and infection on male performance remains unknown.

The *Daphnia‐Pasteuria* system provides an ideal test case for understanding the interplay between host sex, infection, and thermal limits, as each sex exhibits substantial differences in the way they interact with a pathogen. Infected females suffer a severe loss of fecundity (i.e., castration), reduced lifespan, gigantism (Clerc et al., 2015; Hall et al., 2019), and intense competition between pathogen strains when co‐infections occur (Gipson et al., 2019). In contrast, males are more resistant to infection (Duneau et al., 2012), more readily able to limit the proliferation of the pathogen (Gipson et al., 2019; Hall & Mideo, 2018), and suffer lower virulence, in terms of the relative reduction in lifespan, as a result (Gipson & Hall, 2018). Although *Daphnia* populations tend to be female‐biased, as female *Daphnia* reproduce asexually, males can still constitute a large portion of the population when biotic or abiotic stressors are experienced (i.e., pollutants, food stress, pathogens, or changes in photoperiod), including during an outbreak of infectious disease (Duncan et al., 2006; Galimov et al., 2011). With this in mind, we measured the upper thermal limits of males and females in response to static heat stress (Hector et al., 2019), using animals from multiple host and pathogen genotype combinations as part of a standard cross‐infection experiment. We tested whether males, as the host sex more resistant to infection in this species, suffer less from infection in terms of the reduction in their thermal limits, or if all sexes are affected equally when infected by *P. ramosa*.

## 2 MATERIAL AND METHODS

*Daphnia magna* Straus is a small (1–5 mm) planktonic crustacean that reproduces via cyclical parthenogenesis and inhabits brackish and freshwater environments, ranging from shallow pools to lakes (Ebert, 2005). A common pathogen of *D. magna* is the gram‐positive bacteria *Pasteuria ramosa* Metchnikoff, which, as an obligate killer of *D. magna*, is transmitted horizontally after host death (Ebert et al., 2016), but whose epidemiology varies with the sex of its host (Hall & Mideo, 2018). This study utilized genetically identical male and female hosts from two *D. magna* genotypes, BE‐OMZ‐M10 from Belgium (herein M10) and HU‐HO2 from Hungary (herein HO2), and exposed them to one of three novel *P. ramosa* genotypes (C1, C14, and C20) known to vary in their transmission potential and virulence, as well as their impact on a host's thermal limits (Clerc et al., 2015; Hector et al., 2019; Michel et al., 2016). With these chosen genotype combinations, our results are generalizable across a range of typical host and pathogen genetic backgrounds and infectious disease phenotypes.

Before the experiment, the *Daphnia* clones were maintained under standardized conditions for three generations, with animals maintained individually in 70‐ml jars filled with 50 ml of artificial media (ADaM; as per Ebert et al. (2000)) and kept in a single controlled climate chamber (16:8 light:dark cycle and 20°C). Animals were fed daily with green algae (*Scenedesmus* sp., 5 million cells per day as an adult) and transferred into fresh media twice each week. Subsequent experiential animals were maintained under identical conditions.

### 2.1 Generation of male and female *D. magna*

To produce genetically identical males and females, standardized mothers were exposed to a short pulse of the juvenile hormone, methyl farnesoate (Product ID: S‐0153, Echelon Biosciences), following previously established protocols (Thompson et al., 2017). Briefly, mothers were exposed to the hormone at a concentration of 300 μg/L after producing their first clutch and then transferred into fresh hormone‐treated media every 2 days. This treatment encourages the production of male offspring with identical genotypes to their maternal generation (Olmstead & Leblanc, 2002), with no confounding fitness effects on the lifespan, fecundity, infection rates, or spore loads of the offspring (Thompson et al., 2017). All experimental animals were taken from clutches three and four of the hormone‐treated mothers.

### 2.2 Experimental infection and thermal limit assays

Following the generation of male and female *D. magna*, experimental animals were placed individually in glass jars with fresh media (70‐ml jars with 20 ml ADaM) and randomly exposed to one of the three pathogen genotypes, or maintained as an unexposed control. The infection process occurred over 2 days using animals that were four days old, wherein either 20 000 pathogen spores of the appropriate exposure group (C1, C14, and C20) were added daily (40 000 spores total), or for control animals the equivalent volume of a random uninfected *Daphnia* suspension. Three days later, individuals were transferred to clean jars, provided with 50 ml of fresh media, and otherwise maintained under standard conditions (as outlined above) until reaching the age chosen for the thermal limit assay as detailed below (approx. 25 days postinfection based on Hector et al. (2019)). Between 40 and 43 individuals per sex per treatment were initially set up, totaling 665 individuals (2 sexes × 2 host genotypes × 4 infection treatments [pathogen genotype C1, C14, and C20, or uninfected controls] x [40–43] replicates), and approximately equal numbers per treatment were assigned to one of the three experimental blocks.

To assess the thermal limits of each experimental treatment group, a static heat shock assay was performed in which *D. magna* were exposed to 37°C until immobilization occurred, commonly referred to as the knockdown time (as in Hector et al., 2019). Knockdown times measure the capacity of an individual to avoid physical incapacitation during temperature extremes and are a common measure of assessing thermal limits (e.g., Hector et al., 2019; Mitchell & Hoffmann, 2010). A static heat shock temperature of 37°C was chosen following Yampolsky et al. (2014) and is an acute heat stress that is lethal to the animals after several hours or less. Animals were individually placed in 5‐ml glass fly vials covered by a mesh and immersed in an agitated water bath of ADaM solution at the desired temperature of 37°C. The mesh allowed the experimental animals to be contained in the vials while minimizing oxygen depletion over the course of the assays by allowing ADaM to be exchanged between the vials and tank. Time to knockdown was recorded from the time the vials were immersed in the water bath until the cessation of any *Daphnia* movement. Assays were run over 2 days per block using control and infected *D. magna* aged either 25 or 26 days postinfection at the time of the assay.

Following the heat shock assay, the body size of each experimental animal was measured using a stereomicroscope from top of the head above the eye to the base of the tail. For infected animals, spore loads were also quantified using an Accuri C6 Flow Cytometer (BD Biosciences). Animals were first individually homogenized in 500 μl of water. Next, 10 μl of this sample was pipetted into 190 μl of EDTA (5 mM) in a 96‐well PPE plate. Per run, 12 samples were counted with every fourth well containing only EDTA as a wash step. A combination of gates based on fluorescence (via the 670 LP filter) and side scatter (a measure of cell granularity) was then used to quantify the number of mature spores based on their distinct size, morphology, and fluorescence (as opposed to immature spores, algae, or animal debris). Each sample was counted twice, averaged, and then used to estimate the spore load of the infected individual (as per Hector et al., 2019).

### 2.3 Statistical analysis

All statistical analyses were performed in R (ver. 3.0.1; R Development Core Team). Due to differences in infection rates (73%–98%), as well as survival and handling errors, sample sizes for the different treatment combinations varied between 27 and 36 (median 35), with the complete set of data (knockdown times, spore loads, and body size) available for 528 individuals in total (see Table S1). All controls remained uninfected. We first analyzed the knockdown times using a full‐factorial analysis of variance (type III), with host genotype (2 levels: HO2 or M10), host sex (2 levels: male or female), infection treatment (4 levels: pathogen genotype C1, C14, and C20, or uninfected controls), and their interactions as fixed effects. Before analysis, we square‐root‐transformed the raw knockdown times to meet the assumption of normality as assessed using a Shapiro–Wilk test, although the figures are presented on the original scale for ease of interpretation.

We next explored how any change in thermal limits caused by a pathogen might relate to the severity of an infection, as estimated here by the change in body size and the spore load of infected animals, and if this relationship is the same for males and females. To do so, we first calculated the relative change in thermal limits for each infected individual by subtracting from their knockdown time the corresponding control group mean (i.e., sex‐ and genotype‐matched). A series of multifactor analysis of covariance models, with host sex as an interacting factor, were then used to assess how simultaneous changes in spore loads (log‐transformed) and the change in host body size after infection (calculated by subtracting the mean body size of control animals) predict reduction in thermal limits caused by infection. These models estimate the partial effects of increasing spore loads on the changes in thermal limits after controlling for the influence of relative body size (and vice versa) and can assess whether the difference in the relative thermal limits for males and females still persists after correcting for spore loads and relative body sizes. For simplicity, each host and pathogen genotype combination was analyzed separately and the partial effects of spore loads and relative body size on the change in thermal limits visualized for each sex using the *visreg* package (Breheny & Burchett, 2017).

## 3 RESULTS

### 3.1 Infection nullifies any sexual dimorphism in a host's thermal limits

Variation in upper thermal limits was dominated by clear differences between the sexes in their knockdown times, but only when unexposed to any pathogen. In the control animals, the knockdown times of males were significantly less than females for both host genotypes, with a difference of 6 min occurring for genotype M10 (difference: 6.35 ± 1.40 min, sex effect: *F*_1,70_ = 16.58, *p* < .001) and 10 min for genotype HO2 (difference: 9.73 ± 1.04 min, sex effect: *F*_1,69_ = 84.11, *p* < .001). When infected, however, any difference among the sexes in their thermal limits was severely reduced (host genotype M10), or even eliminated (host genotype HO2), contributing to the significant sex by exposure treatment interaction in the linear model describing the overall variability in knockdown times (Table 1).

**TABLE 1:** Results of the three‐factor analysis of variance describing the effects of host genotype, host sex, exposure treatment, and their interactions on the knockdown times of animals exposed to 37°C static heat shock. Presented are the appropriate test statistics and significance levels (α = .05) for each factor and interaction term of the type III analysis of variance (* *p* < .05, ** *p* < .01, *** *p* < .001)

Factor	*F*‐ratio	*df*	*p*‐value	Signif.
Host genotype	7.453	1, 512	.007	***
Host sex	18.305	1, 512	<.001	**
Exposure treatment	59.137	3, 512	<.001	***
Host × sex	7.858	1, 512	.005	**
Host × exposure	10.036	3, 512	<.001	**
Sex × exposure	10.792	3, 512	<.001	***
Host × sex × exposure	0.669	3, 512	.572	

**FIGURE 1:**
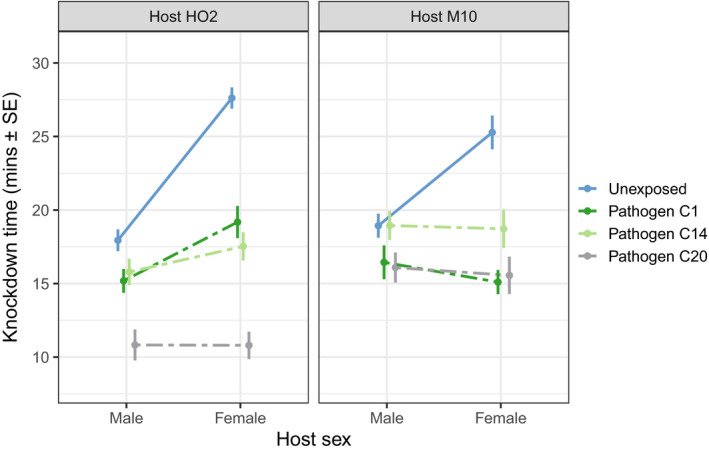
The knockdown times (in minutes) for male and female *Daphnia* exposed to a 37°C static heat shock. Shown are the treatment means and standard errors. Solid lines highlight the difference between males and females of the unexposed control animals, while dashed lines represent the treatments where animals were infected by one of three pathogen genotypes

For host genotype HO2, for example, the average knockdown times for infected animals were only marginally higher in females than males (*F*_1,190_ = 5.59, *p* = .019), with infection by the three pathogen genotypes reducing the thermal limits of males by only 2.3–7.0 min, compared to 8.3–26.8 min in females (see Figure 1 and parameter estimates in Table S2). Similar patterns were seen for host genotype M10, but this time the average knockdown times of infected males and females were indistinguishable (*F*_1,183_ = 0.37, *p* = .444) and the average magnitude of any change in thermal limits was also lower than that observed in host HO2 (0.2–3.0 min loss in males and 6.1–10.1 min loss in females, see Figure 1 and Table S2) compared to host genotype HO2. There were also subtle differences in the rank order for which the pathogen genotypes reduced knockdown times in each host (leading to a significant host by exposure interaction, Table 1), but otherwise, the order of effects remained similar across males and females within a given host genotype.

### 3.2 Females suffer more even after correcting for pathogen performance

Male and female *Daphnia* differ in many characteristics that potentially relate to their capacity to cope with both thermal stress and pathogen infection, as demonstrated here for body size (average size: males 2.39 mm, females 3.50 mm) and the control of pathogen proliferation (average spore production: males 0.92 million, females 3.16 million), and in other studies for traits such as the infection‐induced increase in late‐life mortality (Gipson & Hall, 2018; Gipson et al., 2019) and changes in host movement (Nørgaard et al., 2019). Our results, however, show that the greater reduction in thermal limits that females suffer when infected persists even after correcting for overt sex differences in relative body size and the control of pathogen proliferation. For each host and genotype combination, the analysis of covariance models (Table 2) revealed a strong effect of host sex in all cases (i.e., intercept differences), with similar slopes for the effects of spore loads and relative body size in most cases, as indicated by a general lack of sex by covariate interactions. Only in one combination, pathogen C1 infection of host M10, did we find that the relationship between a covariate (in this case relative body size) and the change in knockdown times varied by sex.

**TABLE 2:** Results of the multivariate analysis of covariance models describing the effects of host sex, spore loads, relative body size, and their interactions on the relative change in knockdown time of infected animals under 37°C static heat shock. Presented are the appropriate test statistics and significance levels (α = .05) for each factor, covariate and interaction terms of analysis of covariance variance models (*p < .05, **p < .01, ***p < .001)

Parameters	Host genotype HO2				Host genotype M10			
	*df*	*F*	*p*‐value	signif.	*df*	*F*	*p*‐value	signif.
Pathogen genotype C1								
Host sex	1, 62	31.854	<.001	***	1, 61	5.393	.024	*
Spore loads	1, 62	9.961	.002	**	1, 61	7.118	.010	**
Relative size	1, 62	1.071	.305		1, 61	0.606	.439	
Sex × spores	1, 62	0.098	.756		1, 61	1.786	.186	
Sex × size	1, 62	1.037	.312		1, 61	4.399	.040	*
Pathogen genotype C14								
Host sex	1, 60	23.230	<.001	***	1, 54	8.127	.006	**
Spore loads	1, 60	5.127	.027	*	1, 54	2.736	.104	
Relative size	1, 60	1.039	.312		1, 54	8.448	.005	**
Sex × spores	1, 60	0.115	.735		1, 54	3.454	.069	
Sex × size	1, 60	1.452	.233		1, 54	2.050	.158	
Pathogen genotype C20								
Host sex	1, 56	8.198	.006	**	1, 56	7.929	.007	**
Spore loads	1, 56	0.552	.461		1, 56	0.223	.639	
Relative size	1, 56	0.691	.409		1, 56	<0.001	.994	
Sex × spores	1, 56	3.806	.056		1, 56	1.296	.260	
Sex × size	1, 56	0.638	.428		1, 56	0.003	.957	

**FIGURE 2:**
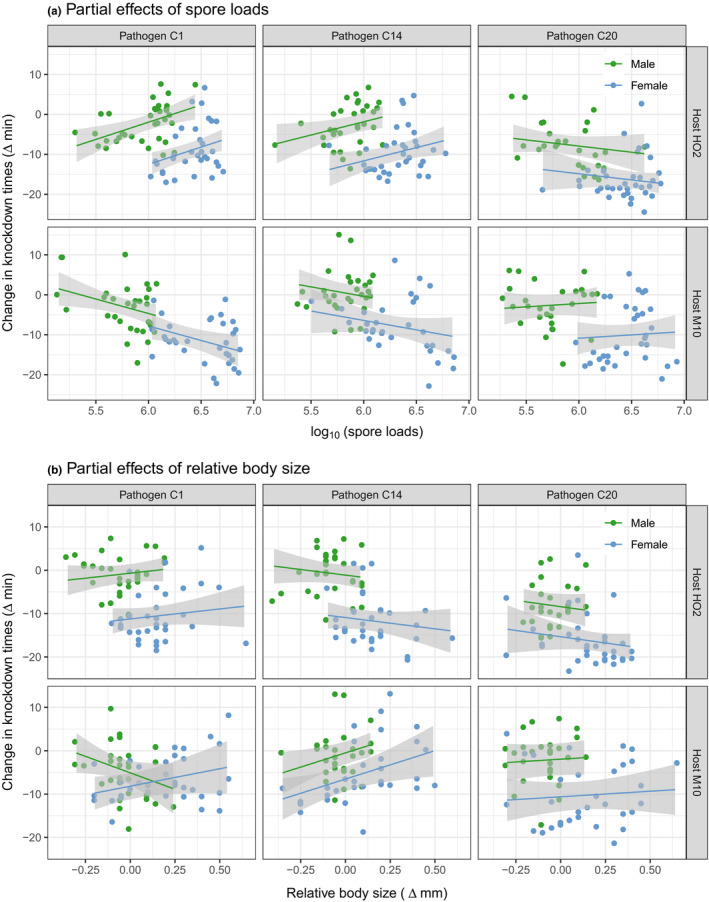
The predicted partial effects of spore loads and relative body size on the change in knockdown times of infected animals. Relative measures of upper thermal limits and body size were calculated by subtracting the corresponding control group means from each individual observation. Shown are the partial residuals and predicted fit (and confidence intervals) for the analysis of covariance models of each host–pathogen combination

The partial effects of spore load and relative body size on the knockdown times of males and females are shown in Figure 2. For spore loads, we observed that changes in load did not significantly predict the knockdown times of infected animals (relative to controls) in three of the six host–pathogen combinations. For the remaining cases, increasing spore loads both positively (pathogen C1 and C14 with host HO2) and negatively (pathogen C1 and host M10) influenced the change in knockdown times. Variation in the relative body size of infected animals was also similarly unrelated to the changes in knockdown times for most host–pathogen combinations. Only in one case did smaller than expected animals suffer the greatest reduction in knockdown times (pathogen C14 and host M10), while in another the response was positive in females and negative in males (pathogen C1 and host M10). Overall, the relationship between spore loads, relative body size, and the change in thermal limits of infected males and females was specific to the host and pathogen genotypic combination in question.

## 4 DISCUSSION

In this study, we examined how host sex and exposure to a pathogen interact to shape the upper thermal limits of an organism. In the absence of any pathogen, our results reinforce how males and females will contribute substantially to variability in the thermal limits of a species. Our finding that females are more heat‐resistant has also been documented, among others, in two species of African mosquito (Lyons et al., 2012) and five of eleven sampled species of *Drosophila* (Mitchell & Hoffmann, 2010, with males being more resistant in only 2 cases). The sexual dimorphism in thermal limits in *Daphnia*, with knockdown times up to 10 min lower for males than females, is comparable to much of the differences observed among populations of *D. magna* (using females only) ranging from latitudes 0° to 66° North (32‐min difference in knockdown times across the entire range, Kenya to the White Sea, Yampolsky et al., 2014). Similar results, pointing to the ecological relevance of sex differences, have also been documented in an Australian *Drosophila* species, whereby the degree of sexual dimorphism, albeit with males now being more heat‐resistant (3 min higher knockdown times on average), is of a similar magnitude to the variability in knockdown times observed along a latitudinal cline from tropical to temperate populations (up to 5‐min difference, Lasne et al., 2018).

The greater thermal resistance of females of both *Daphnia* (as observed here) and other invertebrate species may afford a greater level of protection for these species under upcoming scenarios of global climate change. The higher thermal limits of females, for example, should enable population growth rates to be more readily maintained under thermal stress, given the assumption that female fitness is more tightly linked to population growth than that of males (Crowley, 2000; Harts et al., 2014). When exposed to a pathogen, however, female *Daphnia* suffered a far greater reduction in knockdown times than males once an infection had successfully established (Figure 1). The net result is that the upper thermal limits of females when infected are either indistinguishable from that of males (host M10) or at best now only marginally higher (host HO2). This finding is likely to apply to different forms of heat stress, such as ramping temperature increases, as we have previously shown that the upper thermal limits of different host–pathogen combinations to be highly concordant across a range of temperature changes (Hector et al., 2019). Our results, therefore, highlight how the introduction of a pathogen can potentially negate any buffer that the higher thermal limits of females provide for a population. This effect will be greatest for host–pathogen systems where infection is nonchronic and does not substantially reduce the fecundity of its host, and more limited for systems, such as here with *Pasteuria*, where infection reduces the instantaneous growth rate of the population (but importantly does not drive it to zero, e.g., Auld et al., 2012).

The drastic reduction in knockdown times when females are infected remains even after correcting for the substantial sex differences in the symptoms of infectious disease, such as the higher spore loads and increase in body size (i.e., gigantism) that occurs in females (see also Gipson & Hall, 2018; Gipson et al., 2019; Hall & Mideo, 2018). Female *Daphnia*, therefore, may generally be more resistant to static heat stress, but their upper thermal limits are less tolerant of infection (sensu, Lars et al., 2009), leading to a twofold drop in knockdown times relative to males for a given body size change or spore load (Figure 2). Rather than a clear decline in thermal limits with the proliferation of the pathogen, we observed a range of relationships, both positive and negative, between the reduction in thermal limits and spore loads or relative body size. This contrasts with our previous work, where female *Daphnia*, who were most at risk to thermal stress, had higher spore loads and relatively smaller body sizes (Hector et al., 2019). Yet, unlike Hector et al. (2019), we only measured the thermal limits of individuals at a single age point (25–26 days postinfection). Had the disease progressed further, it is likely that the direct costs of infection by *P. ramosa* would more readily manifest, particularly in females where *P. ramosa* continues to proliferate over the entire course of infection (Clerc et al., 2015; Hall & Mideo, 2018).

In summary, our results reaffirm that males and females can differ substantially in their thermal limits (e.g., Lasne et al., 2018; Lyons et al., 2012; Mitchell & Hoffmann, 2010), but also show for the first time how a pathogen can limit the expression of sexual dimorphism in the thermal limits of its host. As scenarios of global change often include shifts in the distribution or transmission of infectious disease agents (e.g., Altizer et al., 2013; Metcalf et al., 2017), our observations suggest that many species may be at greater risk than previously thought, particularly if the more susceptible sex is also the one on which population growth most depends (e.g., Crowley, 2000; Harts et al., 2014). In *Daphnia*, for example, population growth is strongly tied to female performance, but females are also both more likely to be infected than males (Duneau et al., 2012; Gipson & Hall, 2018) and, as we have shown, suffer a greater reduction in their upper thermal limits as a result. With males and females commonly varying in their susceptibility to infection (Poulin, 1996; Schalk & Forbes, 1997; Zuk, 2009), incorporating sex‐specific responses into an understanding of thermal limits will help determine whether species with separate sexes are more or less vulnerable than previously thought when thermal stress and pathogen exposure are likely to co‐occur.

## CONFLICT OF INTEREST

The authors declare no conflicts of interest.

## AUTHOR CONTRIBUTION

**Tess Laidlaw:** Data curation (lead); Formal analysis (equal); Investigation (lead); Methodology (equal); Visualization (supporting); Writing‐original draft (lead); Writing‐review & editing (supporting). **Tobias E Hector:** Conceptualization (supporting); Investigation (supporting); Methodology (equal); Visualization (supporting); Writing‐review & editing (supporting). **Carla M. Sgro:** Conceptualization (equal); Funding acquisition (supporting); Methodology (supporting); Project administration (supporting); Supervision (equal); Writing‐original draft (supporting); Writing‐review & editing (supporting). **Matthew Hall:** Conceptualization (lead); Data curation (supporting); Formal analysis (equal); Funding acquisition (lead); Investigation (supporting); Methodology (supporting); Project administration (lead); Supervision (equal); Visualization (lead); Writing‐original draft (supporting); Writing‐review & editing (lead).

## Supporting information

Table S1‐S2

## Data Availability

Datasets supporting this article are available at Monash University's repository for research data, https://doi.org/10.26180/5eba128da5310.
