# Supplementary material for: Pathogen exposure reduces sexual dimorphism in a host’s upper thermal limits
Source: Ecol Evol. 2020 Nov 6;10(23):12851–9. doi: 10.1002/ece3.6828 (PMC7713950; doi:10.1002/ece3.6828)
Supplement: Supplementary file 1 — Table S1‐S2 [file ECE3-10-12851-s001.pdf]

Supplementary material:
Pathogen exposure reduces sexual dimorphism in a host's upper thermal limits

Table S1: Sample sizes used in the thermal limit assays (assayed) after accounting for differences in starting numbers (initial), infection rates (infected) and loses due to premature mortality and handling errors (removed).

Host	Sex	Treatment	Initial	Infected	Removed	Assayed
HO2	Male	Control	41	0 (0%)	6	35
		C1	43	41 (95%)	6	35
		C14	42	38 (91%)	6	32
		C20	40	32 (80%)	6	27
	Female	Control	42	0 (0%)	6	36
		C1	41	38 (93%)	5	33
		C14	42	40 (95%)	6	34
		C20	42	41 (98%)	6	35
M10	Male	Control	42	0 (0%)	6	36
		C1	43	35 (81%)	2	33
		C14	41	30 (73%)	1	29
		C20	41	33 (81%)	4	29
	Female	Control	42	0 (0%)	6	36
		C1	40	39 (98%)	5	34
		C14	41	37 (90%)	6	31
		C20	42	39 (93%)	6	33

Table S2: Parameter estimates describing the change in knockdown times associated with each pathogen genotype infection, relative to the control intercept. Models were run separately for each host genotype and host sex combination.

**Parameters**	**Male hosts**			**Female hosts**		
	estimate	SE	*p*-value	estimate	SE	*p*-value
**Host genotype HO2**						
Control intercept	17.94	0.83	< 0.001	27.62	0.91	< 0.001
C1 effect	-2.75	1.17	0.020	-8.44	1.32	< 0.001
C14 effect	-2.15	1.20	0.075	-10.08	1.31	< 0.001
C20 effect	-7.12	1.26	< 0.001	-16.81	130	< 0.001
**Host genotype M10**						
Control intercept	18.93	0.92	< 0.001	25.28	1.11	< 0.001
C1 effect	-2.49	1.35	0.069	-10.18	1.56	< 0.001
C14 effect	-0.02	1.38	0.992	-6.56	1.63	< 0.001
C20 effect	-2.84	1.38	0.041	-9.73	1.60	< 0.001
